# Early Life Inoculation With Adult-Derived Microbiota Accelerates Maturation of Intestinal Microbiota and Enhances NK Cell Activation in Broiler Chickens

**DOI:** 10.3389/fvets.2020.584561

**Published:** 2020-11-19

**Authors:** Nathalie Meijerink, Jannigje G. Kers, Francisca C. Velkers, Daphne A. van Haarlem, David M. Lamot, Jean E. de Oliveira, Hauke Smidt, J. Arjan Stegeman, Victor P. M. G. Rutten, Christine A. Jansen

**Affiliations:** ^1^Division Infectious Diseases and Immunology, Department Biomolecular Health Sciences, Faculty of Veterinary Medicine, Utrecht University, Utrecht, Netherlands; ^2^Division Farm Animal Health, Department Population Health Sciences, Faculty of Veterinary Medicine, Utrecht University, Utrecht, Netherlands; ^3^Laboratory of Microbiology, Wageningen University & Research, Wageningen, Netherlands; ^4^Cargill Animal Nutrition and Health Innovation Center, Velddriel, Netherlands; ^5^Cargill R&D Center, Vilvoorde, Belgium; ^6^Department of Veterinary Tropical Diseases, Faculty of Veterinary Science, University of Pretoria, Pretoria, South Africa

**Keywords:** poultry, avian immunology, intestinal microbiota, intraepithelial lymphocytes, innate immunity, NK cells

## Abstract

Studies in mammals, including chickens, have shown that the development of the immune system is affected by interactions with intestinal microbiota. Early life microbial colonization may affect the development of innate and adaptive immunity and may contribute to lasting effects on health and resilience of broiler chickens. We inoculated broiler chickens with adult-derived-microbiota (AM) to investigate their effects on intestinal microbiota composition and natural killer (NK) cells, amongst other immune cells. We hypothesized that AM inoculation directly upon hatch (day 0) would induce an alteration in microbiota composition shortly after hatch, and subsequently affect (subsets of) intestinal NK cells and their activation. Microbiota composition of caecal and ileal content of chickens of 1, 3, 7, 14, 21, and 35 days of age was assessed by sequencing of 16S ribosomal RNA gene amplicons. In parallel, subsets and activation of intestinal NK cells were analyzed by flow cytometry. In caecal content of 1- and 3-day-old AM chickens, a higher alpha-diversity (Faith's phylogenetic diversity) was observed compared to control chickens, whereas ileal microbiota were unaffected. Regarding beta-diversity, caecal microbiota profiles could be clustered into three distinct community types. Cluster A represented caecal microbiota of 1-day-old AM chickens and 1- and 3-day-old control chickens. Cluster B included microbiota of seven of eight 3- and 7-day-old AM and 7-day-old control chickens, and cluster C comprised microbiota of all chickens of 14-days and older, independent of inoculation. In 3-day-old AM chickens an increase in the percentages of intestinal IL-2Rα^+^NK cells and activated NK cells was observed compared to control chickens of the same age. In addition, an increase in relative numbers of intestinal cytotoxic CD8αα^+^T cells was observed in 14- and 21-day-old AM chickens. Taken together, these results indicate that early exposure to AM shapes and accelerates the maturation of caecal microbiota, which is paralleled by an increase in IL-2Rα^+^NK cells and enhanced NK cell activation. The observed association between early life development of intestinal microbiota and immune system indicates possibilities to apply microbiota-targeted strategies that can accelerate maturation of intestinal microbiota and strengthen the immune system, thereby improving the health and resilience of broiler chickens.

## Introduction

Health and production efficiency of broiler chickens are of major importance, as chicken meat is a key sustainable source of animal protein for the growing human population (1, 2). In poultry production, restrictions of the use of antimicrobials have made other strategies to maintain or improve poultry health, such as enhanced immune responsiveness, increasingly important.

A crucial role in chicken health and production performance is played in many physiological processes by intestinal microbiota, including nutrient digestion and absorption, metabolism, intestinal barrier function, and development of intestinal immunity (3, 4). The maturation of the intestinal microbiota of chickens entails rapid successional changes, developing from a simple, to a more complex and diverse composition due to gradual colonization with microbiota (5–7). Early life exposure to microbiota is an important driver of this development, which can also affect health later in life. This has been shown in human infants (8–10), and other mammals and hatchlings treated with antibiotics early in life or raised under extreme hygienic conditions, e.g., germ-free or SPF environments (11–15). Also, in commercial chickens under normal circumstances, early transiently colonizing bacteria have been shown to have a large effect on intestinal microbiota composition later in life (16–18). However, due to hatching in a hatchery environment, colonization in commercial chickens starts with microbiota from environmental, rather than parental sources. As these environmental microorganisms may include pathogenic bacteria, competitive exclusion products derived from intestinal microbiota of healthy adult chickens have been developed to compete with colonization by pathogenic bacteria and are widely used in poulty production systems to induce a healthy microbiota (19). When supplied *in ovo* or to hatchlings, adult-derived microbiota has been shown to accelerate bacterial colonization (20–22) and to decrease the occurrence of undesirable bacteria such as *Salmonella* and *Escherichia coli* (19, 23, 24).

The intestinal immune system plays an important role in the defense against pathogens that enter a host via the gut. Underneath the mucus layer [the first protective barrier in the intestinal tract (25)], a layer of epithelial cells including immune cells such as the intraepithelial lymphocytes (IEL) is observed. The population of IEL consists of high numbers of γδ T cells, adaptive CD8^+^ T cells and innate natural killer (NK) cells (26). During embryonic development and early life, when resistance against pathogens relies on innate immune responses since the adaptive immune system is not yet fully developed, NK cells are important players (27, 28). Chicken NK cells have also been reported in multiple organs including the intestine, lung, spleen, and blood (26, 29, 30). Previously, we and others showed that a high percentage of intestinal NK cells in chickens are recognized by the marker 28-4 (26), which was identified as CD25 or IL-2Rα (26). In mammals, the IL-2Rα chain is expressed on NK cells early upon activation (31), and this is followed by enhanced NK cell mediated killing and IFNγ production (31). Another marker found to be expressed on intestinal NK cells was 20E5 (32). It is also expressed on cells that show NK cell activation (29). Furthermore, elsewhere in the body, increased surface expression of CD107 indicative of NK cell activation was observed on primary chicken NK cells in lung, spleen and blood upon infections with avian viruses (30, 33, 34).

In the intestinal tract many interactions occur between the microbiota and immune cells (35, 36). These interactions are important for the development of the immune system, as was shown in mammals (21, 37, 38) and chickens (14, 39). For example, early life transplantation of adult microbiota has resulted in increased natural antibody titers in laying chickens (40) paralleled by long lasting effects on mRNA levels of pro-inflammatory cytokines (41). Disturbing the early life microbiota in 1-day-old broiler chickens by antibiotics resulted in reduced numbers of macrophage-like cells in the jejunum (14), whereas differences in rearing environment, e.g., a reduction in environmental microbial exposure resulted, in two phylogenetically distinct lines of broiler chickens, in lower expression levels of β-defensins (42).

Studies in rodents and humans have shown that specific probiotic microorganisms enhance intestinal NK cell activity and cytokine production (43) either directly via their interaction with receptors expressed on NK cells (44, 45), or indirectly via cytokine production of resident myeloid or epithelial cells (46). Also the adaptive immune system can be modulated via interactions with the microbiota (47–50), or indirectly through innate immune cell activities. As other studies in rodents and humans have shown, the microbiota affects activation of γδ T cells (51, 52) and CD8^+^ T cells (53). Taken together, this indicates that the composition and activity of the microbiota and its effects on the immune system in early life may have long term consequences on the health of individuals.

In chickens, previous studies addressed the effect of microbiota on innate immune responses in the intestine, spleen and blood by studying mRNA levels of immune related genes (41, 42) by immunohistochemistry (14) and by analysis of natural antibody titers (40). In this study, we used tools that we developed previously for the analysis of the phenotype and the function of chicken innate immune cells (29, 54) to assess whether and to what extent differences in early life microbial colonization would affect the development of NK cells locally (in the intestine) and systemically (in spleen and blood).

We hypothesized that inoculation with adult-derived microbiota(AM) upon hatch would induce an alteration in microbiota development and affect the presence and activation of intestinal NK cells. To induce early colonization with a rich, complex microbiota to stimulate immune development, we used Aviguard® (MSD Animal Health, the Netherlands), as this product derived from microbiota of healthy adult chickens has been shown to be able to colonize the intestinal tract and induce early maturation of the intestinal microbiota in previous studies with hatchlings (22, 55). In this study, AM inoculation resulted in an accelerated maturation of the intestinal microbiota, an increase of IL-2Rα^+^ NK cells and enhanced activation of NK cells. The observed association between early life development of intestinal microbiota and the immune system indicates possibilities to apply microbiota-targeted strategies that can accelerate maturation of intestinal microbiota and strengthen the immune system to improve the health and resilience of broiler chickens.

## Materials and Methods

### Birds and Husbandry

Ross 308 broiler 17- and 18-day old embryonated eggs were obtained from the same parent flock of a commercial hatchery (Lagerwey, the Netherlands). ED17 (hatch group A, *n* = 52) and ED18 eggs (hatch group B, *n* = 52) were disinfected with 3% hydrogen peroxide and placed in disinfected egg hatchers. All eggs hatched at ED21. Directly upon hatch, chickens (day 0 in age) were randomly divided into two treatment groups, weighed, labeled and inoculated. Next, the chickens of the two treatment groups were placed in separate floor pens of 2 × 1.5 m (pens 1 and 2), with a solid wall separating the pens. Each pen was divided in two equal parts of 1 × 1.5 m for chickens from hatch group A and B. The pens were lined with wood shavings (2 kg/m^2^, sterilized by autoclavation). Non-sterilized standard commercial starter and grower feeds (Research Diet Services, the Netherlands) and water was provided *ad libitum*. No antibiotics, coccidiostatic drugs or commercial vaccines were applied during the experiment. A standard lighting, temperature scheme for Ross broiler chickens was used, and conditions were kept the same for all compartments. The chickens were observed daily for clinical signs, abnormal behavior or mortality and were also evaluated for presence of abnormalities during post-mortem. No signs of disease or impaired health were observed in both groups throughout the experiment. Feed intake and body weight were assessed in both groups at each sampling moment and followed the expectations based on the Ross 308 broiler performance standards in both groups.

The experimental room was equipped with a mechanical negative pressure ventilation system.

The animal experiment was approved by the Dutch Central Authority for Scientific Procedures on Animals and the Animal Experiments Committee (registration number AVD1080020174425) of Utrecht University (the Netherlands) and all procedures were done in full compliance with all relevant legislation.

### Experimental Design

Chickens were inoculated once immediately after hatch to reduce opportunities for prior exposure to microbiota. First, the control group received an oral inoculation with 0.5 ml PBS (Lonza, Basel, Switzerland). The other group, henceforth referred to as the AM group, was inoculated with 0.5 ml of PBS containing 0.05 g/ml of competitive exclusion product Aviguard® (MSD Animal Health, the Netherlands). This is a freeze-dried powder, soluble in water, consisting of fermented, undefined cultures from intestinal microbiota of healthy specific-pathogen-free birds and was used according to manufacturer's instructions. To determine the microbial composition of the AM inoculum and compare this to the microbiota in the chickens, four aliquots of 2 ml were stored at −80°C for DNA extraction. The experimental design of the study is shown in Supplementary Figure 1.

### Sample Collection

At day 0 (upon hatch), four non-inoculated chickens per hatch group were randomly selected and sacrificed, to collect caecal and ileal content for microbiota analyses, as has been described in (56). Ileal content was collected distal and close to the Meckel's diverticulum. The intestinal content was gently squeezed into a 2 ml sterile cryotube, snap frozen on dry ice and stored at −80°C for DNA extraction. The time between sacrificing and placing the intestinal samples on dry ice was between 3–5 min. To avoid cross contamination, all management and biotechnical procedures were completed first with the control group and for each compartment at the same time. At days 1 (24 h after inoculation), 3, 7, 14, 21, and 35, eight chickens (four from the control and four from the AM group) were randomly selected per hatch group (A/B) and sacrificed to collect caecal and ileal content as described above. At day 0 and day 1, the chickens were too small to collect sufficient cells for immunological analyses. Therefore, ileum tissue, spleen and blood were collected from day 3 onwards from six of these eight chickens (*n* = 3 per hatch group). All chickens were weighed prior to post-mortem analyses.

### DNA Extraction

In total, 104 caecal and 104 ileal content samples, consisting of 52 samples per treatment group, and four samples of the AM inoculum were analyzed for microbiota composition. DNA was extracted from 0.25 g content, using 700 μl of Stool Transport and Recovery (STAR) buffer (Roche Diagnostics Nederland BV, the Netherlands). All samples were transferred to sterile screw-capped 2 ml tubes (BIOplastics BV, the Netherlands) containing 0.5 g of zirconium beads (0.1 mm; BioSpec Products, Inc., USA), and 5 glass beads (2.5 mm; BioSpec Products). All samples were treated in a bead beater (Precellys 24, Bertin technologies, France) at a speed of 5.5 m/s for 3 × 1 min, followed by incubation at 95°C with agitation (15 min and 300 rpm). The lysis tube was centrifuged (13,000 g for 5 min at 4°C), and the supernatant was transferred to a 2 ml microcentrifuge tube. Thereafter, the above-described process was repeated with 300 μl STAR buffer. An aliquot (250 μl) of the combined supernatants from the sample lysis was then transferred into the custom Maxwell® 16 Tissue LEV Total RNA Purification Kit cartridge. The remainder of the extraction protocol was then carried out in the Maxwell® 16 Instrument (Promega, the Netherlands) according to the manufacturer's instructions. DNA concentrations were measured with a NanoDrop ND-1000 spectrophotometer (NanoDrop® Technologies, DE, USA), and the DNA samples were stored at −20°C until further use.

### qPCR, 16S rRNA Gene Amplification, Sequencing, and Data Processing

Extracted DNA was diluted to 20 ng μl^−1^ in nuclease free H_2_O. All PCR plastics were UV irradiated for 15 min before use. To validate the AM inoculation, absolute quantification of the bacterial 16S rRNA genes by real-time PCR amplification was performed for the caecal content samples of day-old chickens. For ileal content samples the amount of DNA was too low to reliably determine gene copy numbers. All qPCR assays (CFX384™ real-time PCR detection system, Bio-Rad, Hercules, CA, USA) were performed in triplicate with 25 μl reactions and was described previously (57). For 16S ribosomal RNA (rRNA) gene-based microbial composition profiling, barcoded amplicons covering the variable regions V5–V6 of the bacterial 16S rRNA gene were generated by PCR using the 784F and 1064R primers as described before (58). Each sample was amplified in duplicate using Phusion hot start II high fidelity polymerase (Finnzymes, Finland), checked for correct size and concentration on a 1% agarose gel and subsequently combined and purified using CleanNA magnetic beads (CleanNA, the Netherlands). A detailed description of the PCR conditions is given elsewhere (56). Positive and negative controls were added to the data set to ensure high quality sequencing data. As positive controls we used synthetic mock communities of known composition (58), and as negative controls we used nuclease free water. The resulting libraries were sent to Eurofins Genomics GmbH (Germany) for sequencing on an Illumina Hiseq2500 instrument. The 16S rRNA data was analyzed using NG-tax 2.0 (59). In short, paired-end libraries were filtered to contain only read pairs with a perfect match to the primers and perfectly matching barcodes, to demultiplex reads by sample. Amplicon sequence variants (ASVs) were defined as unique sequences. The ASV picking strategy was based on a *de novo* reference approach. Taxonomy was assigned using the SILVA 128 16S rRNA gene reference database (60). Caecal content samples of day 0 and ileal content samples of day 0 and 1 were excluded from the analysis, because these contained a large number of families associated with the negative control samples, and therefore did not pass our quality control standards. Raw sequence data were deposited into the Sequence Read Archive (SRA) at NCBI under accession number PRJNA670739.

### Isolation of Tissues and Cells

Ileum segments (±10 cm distal from Meckel's diverticulum), spleens and blood (5 ml) were collected. Ileum segments were washed with PBS to remove contents and cut in sections of 1 cm^2^ and washed again. Subsequently, IELs were collected by incubating three times in EDTA-medium [HBSS 1x (Gibco BRL) supplemented with 10% heat-inactivated FCS (Lonza); 1% 0.5M EDTA (Sigma-Aldrich)] at 200 rpm for 15 min at 37°C. Supernatants were collected and centrifuged 5 min at 1,200 rpm at 20°C. Cells were then resuspended in PBS, lymphocytes were isolated using Ficoll-Paque Plus (GE Healthcare, the Netherlands) density gradient centrifugation for 12 min at 1,700 rpm, washed in PBS using centrifugation for 5 min at 1,300 rpm and resuspended at 4.0 × 10^6^ cells/ml in NK medium [IMDM supplemented with 8% heat-inactivated FCS (Lonza); 2% heat-inactivated chicken serum, 100 U/ml penicillin/ streptomycin, and 2 mM glutamax I; Gibco BRL, United Kingdom]. Spleens were homogenized using a 70 μm cell strainer [Beckton Dickinson (BD) Biosciences, NJ, USA] to obtain a single cell suspension. Next, lymphocytes in spleen and blood were isolated by Ficoll-Paque density gradient centrifugation (20 min at 2,200 rpm), washed in PBS and resuspended at 4.0 × 10^6^ cells/ml in NK medium as described for ileum.

### Flow Cytometry

Presence and activation of NK and T cell subsets were determined in IEL, spleen, and blood. Unless described otherwise, all antibodies were obtained from Southern Biotech (AL, USA). Markers known to be expressed on chicken NK cells (hybridomas provided by Göbel), such as mouse-anti-chicken-28-4 (IL-2Rα; IgG3) and−20E5-BIOT (IgG1) were co-stained with mouse-anti-chicken-CD45-FITC (IgM) and -CD3-APC (CT3; IgG1) mAb to exclude T cells. The T cell panel included the following markers: mouse-anti-chicken-CD3-PE (CT3; IgG1), -CD4-APC (CT4; IgG1), -TCRγδ-FITC (TCR-1, IgG1), -CD8α (EP72, IgG2b), and -CD8β-BIOT (EP42, IgG2a). Secondary antibody staining was performed using goat-anti-mouse-IgG3-PE and streptavidin-PercP (BD Biosciences) in the NK cell panel, and goat-anti-mouse-IgG2b-APC/Cy7 and streptavidin-PercP in the T cell panel. To asses CD107 expression on NK cells, lymphocytes were washed in PBA and stained with mouse-anti-chicken-CD3-PE, -TCRγδ-BIOT (TCR-1, IgG1),−28-4, and -CD41/61-FITC (11C3, IgG1, Serotec) to exclude thrombocytes from analysis. Secondary antibody staining was performed using streptavidin-PercP and goat-anti-mouse-IgG3-APC/Cy7. All staining procedures were incubated for 20 min at 4°C in the dark, washed in PBA and subsequently stained with a live/dead marker (Zombie Aqua™ Fixable Viability Kit, Biolegend) for 15 min at RT in the dark to exclude dead cells. Finally, lymphocytes were fixed using 2% paraformaldehyde (Merck, Germany) for 10 min at RT, washed and resuspended in 200 μl PBA. Fluorescence of cells was assessed in 150 μl or 50,000 lymphocytes in the live gate using a FACSCANTO II Flowcytometer (BD Biosciences), and data was analyzed with software program FlowJo (Tree star Inc, OR, USA).

### NK Cell Activation Assay

NK cell activation was determined using the CD107-assay, which measures increased surface expression of CD107 as a result of degranulation of perforin and granzymes (29). Briefly, lymphocytes isolated from IEL, spleen, and blood were resuspended in NK medium, and 1 × 10^6^ lymphocytes per sample were used. Lymphocytes were cultured in presence of 1 μl/ml Golgistop (BD Biosciences) and mouse-anti-chicken-CD107-APC mAb (5G10, IgG1, hybridomas provided by Göbel, T.W., Ludwig Maximilians University, Germany) during 4 h at 37°C, 5% CO_2_. Next, cells were washed, stained with monoclonal antibodies and analyzed by flow cytometry.

### Data Analysis

Statistical analyses for microbiota and the relation between microbiota and the immune system were performed in R version 3 (R Foundation for Statistical Computing, Austria), using the packages Phyloseq, Microbiome, Vegan, and DirichletMultinomial (61–64). A Kruskal-Wallis test was used to test for difference in 16S rRNA gene counts in caecal content of day-old chickens between treatment groups.

Alpha and beta diversity metrics and multivariate statistical analyses were applied to determine differences in the measured intestinal microbiota between the two treatment groups and with age. The alpha diversity (within sample) data was determined using Faith's phylogenetic diversity. Faith's phylogenetic diversity not only takes the number of different taxa (ASVs) into account, but also the phylogenetic relatedness of these taxa (65). To test for differences in relative abundance of genera between treatment groups, we used a Wilcoxon rank-sum test and corrected for multiple comparisons using the Benjamini-Hochberg (BH) procedure. The beta diversity (between samples) was determined using weighted and unweighted UniFrac metrics (66). Multivariate microbiota data were visualized using principal coordinates analysis (PCoA, multidimensional scaling method), and non-parametric permutational analysis of variance (PERMANOVA) tests were used to analyze group differences within multivariate community data (67).

To assess whether the development of the microbiota proceeded through different stages of maturation in the two treatment groups, Dirichlet Multinomial Mixtures (DMM) modeling was applied, using a probabilistic model, to identify possible clusters (types) of microbial composition 16S rRNA gene sequence data (68) based on the relative abundance of the microbial groups at genus level. Two separate DMM models were used to study clustering of the microbiota data of the caecal content and ileal content separately. Next, to test whether the observed differences in the microbial development between treatments were associated with differences in immune development, Wilcoxon rank-sum test, corrected for multiple comparisons using BH, was used to test for associations between the identified DMM clusters of microbial composition and immunological parameters. As ileal microbiota clustering did not indicate differences in microbial development between treatments, only the clusters identified for the caecal microbiota profiles were used. Associations were tested for a subset of immunological parameters that showed differences between AM and control chickens of the same age. Furthermore, parameters with fewer than four observations per treatment group and day of age were omitted. The final selection of parameters included percentages and absolute numbers of intestinal IL-2Rα^+^, 20E5^+^ and CD107^+^ NK cells, and CD8αα^+^ T cells.

Statistical analyses for the immunological parameters were done with GraphPad Prism 7.0 software (GraphPad Software Inc., USA), using the Mann-Whitney *U*-test to test differences between treatment groups at a specific day of age. A *p*-value of <0.05 was considered statistically significant.

## Results

### AM Treatment Influences the Composition and Development of the Intestinal Microbiota in Newly Hatched Chickens

The total bacterial 16S rRNA gene copy numbers 24 h after inoculation were significantly higher in caecal content samples at day 1 in AM inoculated compared to control chickens, indicating the presence of a higher quantity of bacteria after inoculation with AM (Supplementary Figure 2).

**Figure 1:**
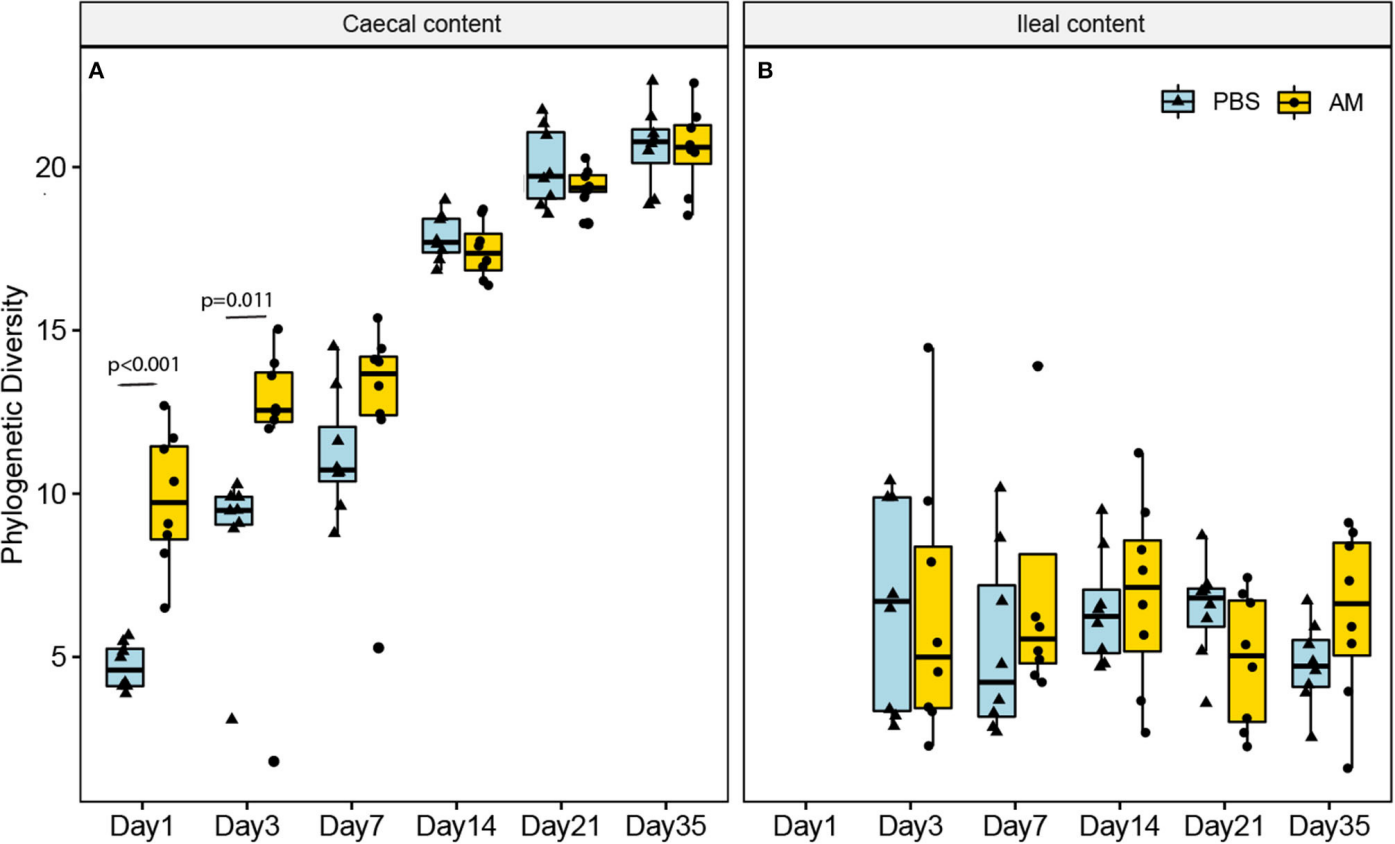
Phylogenetic diversity of the ileal and caecal content microbiota at different ages. **(A)** The phylogenetic diversity (alpha diversity, at ASV level) was only significantly higher in the caecal content of AM chickens compared to controls on day 1 and day 3 (*p* < 0.05). **(B)** In the ileal content microbiota no differences were observed at any of the ages. *n* = 8 chickens per treatment per day of age, whiskers show 95% interval, box 50% interval.

To investigate the effect of AM inoculation on the microbiota composition at different ages in the broiler chickens, alpha and beta diversities, as well as differences in relative abundance of individual microbial taxa, were assessed. The phylogenetic diversity metric, providing information on the number as well as phylogenetic relatedness of observed microbial taxa at the ASV level, was used as an alpha diversity measure to determine differences between AM and control chickens. The phylogenetic diversity of the caecal content was higher in 1- and 3-day-old AM chickens compared to controls, but not for any of the other ages (Figure 1A). In contrast, the phylogenetic diversity of ileal content microbiota did not differ between treatment groups at any age (Figure 1B).

**Figure 2:**
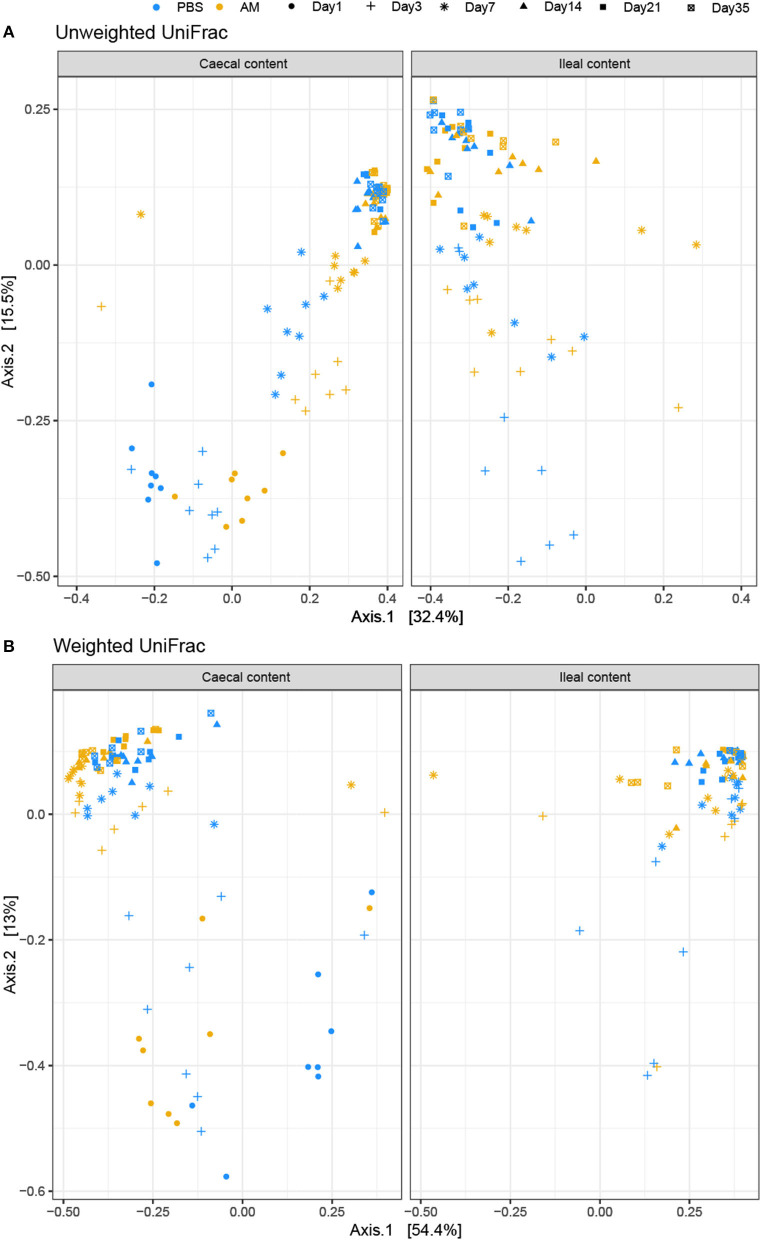
PCoA plot visualizing caecal and ileal content microbial profiles. Unweighted **(A)** and weighted **(B)** UniFrac distance based PCoA on caecal (left) and ileal (right) content samples. **(A)** PERMANOVA of caecal content microbiota showed that treatment explained 6–9% of the variation in caecal microbiota composition between samples (*p* < 5e-04; unweighted UniFrac, *p* < 2e-04, weighted UniFrac), whereas age explained 49–41% of the variation between samples (*p* < 5e-04; unweighted UniFrac, *p* < 2e-04, weighted UniFrac). **(B)** PERMANOVA of ileal content samples showed that treatment explained 4% of the variation in ileal microbiota composition based on unweighted UniFrac, whereas treatment did not significantly contribute to explaining the observed variation using the weighted UniFrac distance metrics (*p* = 0.038; unweighted UniFrac, *p* = 0.355, weighted UniFrac). *n* = 8 chickens per treatment per day of age.

Beta diversity, i.e., the similarity in composition between samples, was determined using the weighted and unweighted UniFrac distance metrics to determine the influence of age and treatment on the composition. Two dimensional visualization of the caecal content microbiota profiles in PCoA plots placed 3- and 7-day-old AM inoculated chickens closely together, indicating high similarity in microbiota composition between these age groups (Figure 2). PERMANOVA of caecal content microbiota showed that treatment explained 6–9% of the variation in caecal microbiota composition between samples (*p* < 5e-04; unweighted UniFrac, *p* < 2e-04, weighted UniFrac), whereas age explained 49–41% of the variation between samples (*p* < 5e-04; unweighted UniFrac, *p* < 2e-04, weighted UniFrac). PERMANOVA of ileal content samples showed that treatment explained 4% of the variation in ileal microbiota composition based on unweighted UniFrac, whereas treatment did not significantly contribute to explaining the observed variation using the weighted UniFrac distance metrics (*p* = 0.038; unweighted UniFrac, *p* = 0.355, weighted UniFrac, Figure 2B), indicating that differences in microbial profiles of ileal samples between treatment groups concerned mostly the presence/absence of taxa occurring at low relative abundance. Age explained 29–24% of the variation between the ileal content samples (*p* < 1e-04; unweighted UniFrac, *p* < 1e-04, weighted UniFrac).

In the AM inoculum 24 different genera were detected, for which the relative abundances in caecal and ileal samples were compared between AM and control chickens. A higher relative abundance in caecal content of AM chickens compared to controls was found for 10 of these 24 genera at day 1, five on day 3, four at day 7, and two at day 14 and 21. At day 35 none of these genera differed in relative abundance between AM and control chickens (Table 1). This indicates that AM inoculation had an impact on the relative abundance of genera at an early age, but did not permanently influence the relative abundance of these genera in the caecal content samples. For ileal content, no differences in the relative abundances of the 24 genera of the inoculum were observed at any of the different ages (data not shown).

**Table 1:** Relative abundance of genera present in the AM inoculum and differences in relative abundance in caecal content for AM compared to control chickens.

**Relative abundance AM inoculum**			**Differences in relative abundance AM vs. control chickens**														
			**Day 1**			**Day 3**			**Day 7**			**Day 14**			**Day 21**		
**Genera**	**RA%**	**SD%**	**AM%**	**PBS%**	***P***	**AM%**	**PBS%**	***P***	**AM%**	**PBS%**	***P***	**AM%**	**PBS%**	***P***	**AM%**	**PBS%**	***P***
*Eubacterium coprostanoligenes group*	0.65	0.22	-	-		0.06	-		-	-		0.84	1.12		0.71	0.65	
*Bacteroides*	0.47	0.06	-	-		3.91	-	**0.045**	2.57	-	**0.018**	2.01	1.12		3.01	3.57	
*Blautia*	0.30	0.09	9.17	-	**0.006**	5.86	3.92		4.67	16.63		4.35	13.14		6.10	10.68	
*Candidatus_Soleaferrea*	0.39	0.06	0.56	-	**0.006**	-	-		-	-		-	-		-	-	
*Clostridium sensu stricto 1*	2.77	0.45	24.32	53.93	**0.033**	1.96	22.30		-	0.19		-	-		0.03	-	
*Clostridium sensu stricto 2*	0.72	0.12	0.77	-	**0.006**	-	-		-	-		-	-		-	-	
*Collinsella*	0.53	0.07	0.68	-	**0.034**	4.65	-	**0.018**	3.64	1.04		1.54	4.30		3.60	2.02	
*Enterococcus*	10.80	1.07	10.12	17.64		16.19	18.55		0.86	0.87		0.32	0.36		0.43	0.48	
*Erysipelatoclostridium*	2.53	0.09	0.26	0.00		2.56	-	**0.027**	0.05	2.07	**0.018**	0.81	2.01		0.44	1.06	**0.043**
*Escherichia-Shigella*	0.57	0.02	32.73	3.36	**0.006**	0.72	11.07	**0.044**	0.16	0.73		-	0.03		-	-	
*Eubacterium*	0.66	0.04	0.30	-	**0.016**	0.46	0.11		0.92	0.28		0.15	0.19		0.07	0.12	
*Flavonifractor*	1.02	0.14	1.23	-	**0.006**	0.90	0.66		0.13	0.44		0.05	0.43	**0.010**	-	-	
*Lachnoclostridium*	9.78	0.93	2.30	-	**0.006**	3.28	-	**0.018**	0.77	0.85		0.66	0.72		0.41	0.16	
*Lactobacillus*	14.96	1.33	8.46	-		6.83	1.12		8.05	3.44		5.34	12.34		13.85	10.14	
*Megamonas*	1.55	0.56	0.02	-		4.05	-		30.21	-	**0.018**	27.46	-	**0.009**	7.46	-	**0.022**
*Megasphaera*	3.30	0.74	-	-		-	-		-	-		-	-		-	-	
*Negativicoccus*	3.62	0.66	-	-		-	-		-	-		-	-		-	-	
*Oscillibacter*	1.94	0.18	-	-		-	-		-	-		-	-		-	-	
*Peptostreptococcus*	30.97	4.04	0.19	-	**0.034**	**-**	**-**		-	-		**-**	-		-	-	
*Sellimonas*	1.31	0.38	-	-		**-**	**-**		0.31	0.75		0.60	0.84		0.50	0.88	
*Slackia*	0.34	0.09	0.02	-		0.03	-		0.43	-	**0.037**	0.01	0.05		0.08	0.10	
*Sutterella*	1.76	0.21	-	-		-	-		-	-		-	-		-	-	
Uncultured	4.45	3.56	0.00	0.00		1.40	0.27		1.07	2.15		1.37	1.96		1.11	1.48	
unknown	0.08	0.09															

*The AM inoculum contained 24 different genera and the genera for which significant differences in relative abundance of AM inoculated chickens (AM) vs. controls (PBS) were found for day 1, 3, 7, 14, and 21 are indicated in bold (n = 8). No differences were observed between treatments on day 35. Results are based on differences of relative abundance tested with Wilcoxon rank-sum test. P = adjusted p-values (<0.05) were corrected for multiple testing with Benjamini-Hochberg (BH). - = not detected. RA = Relative abundance (%) in the AM inoculum. AM/PBS = Relative abundance (%) in AM/control treatment*.

**Figure 3:**
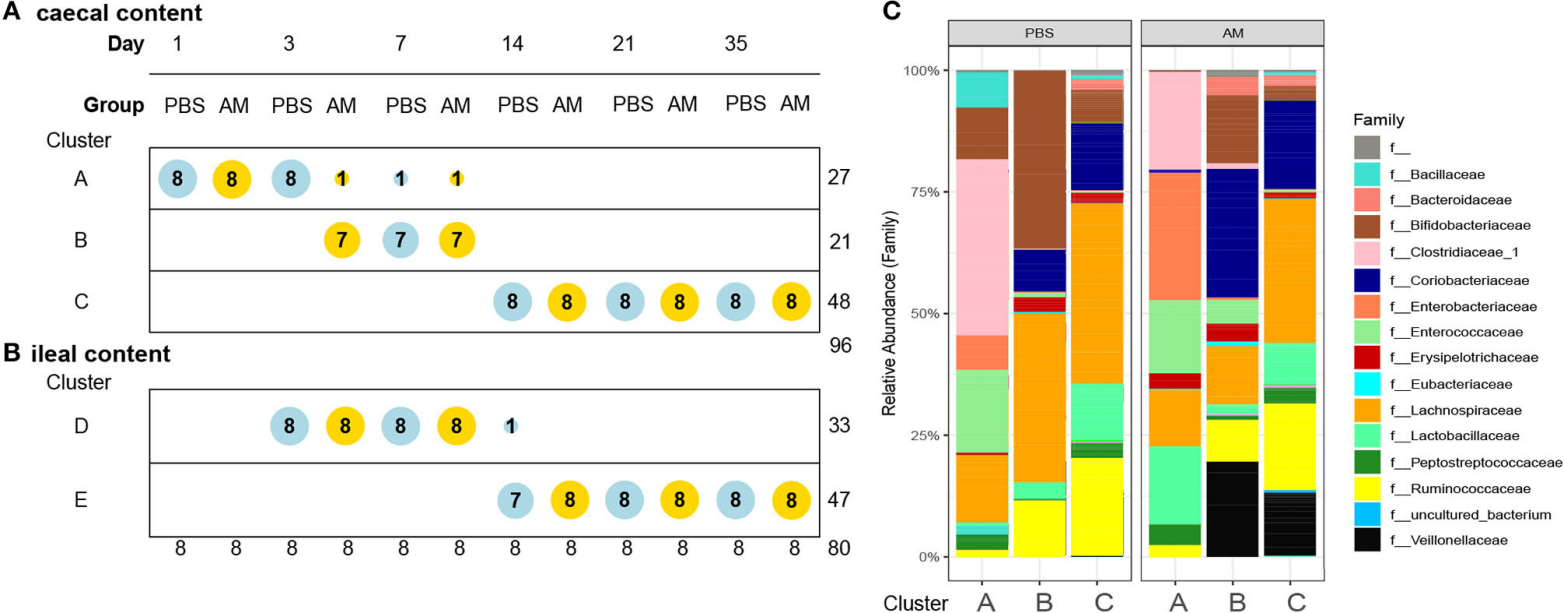
Dirichlet multinomial mixtures (DMM) clustering of 16S rRNA gene sequencing data for caecal and ileal microbial profiles. **(A)** DMM clustering showed the best model fit for three clusters in the caecal content profiles (lowest Laplace approximation, *n* = 96). Cluster A contains 27 samples, Cluster B 21 samples, and the remaining 48 samples are in cluster C. Cluster B contains 3-day old AM chickens, and seven of eight AM and all control chickens of day 7, indicating acceleration of microbiota maturation in the caecal content. Nodes are colored according to intervention (AM or PBS) and ordered according to age. **(B)** In the ileal content samples two distinct clusters were observed, but no evidence for acceleration of the development of the microbiota (*n* = 80). **(C)** Relative microbial abundance of the clusters observed in the caecal content stratified by the intervention at family level.

To assess if AM inoculation affected the development of the microbial composition from hatch toward a mature microbiota, microbial profiles were subjected to DMM clustering of 16S rRNA gene sequencing data based on the relative abundance of microbial taxa at genus level. The DDM method showed the best model fit, based on lowest Laplace approximation, for three clusters in the caecal content profiles (Figure 3A). Cluster A contained 26 samples, with all 1-day-old AM and control chickens and all 3-day-old controls. Cluster B consisted of 21 samples, containing seven of the eight 3-day-old AM chickens and 7-day-old AM and control chickens. The remaining 48 samples were in cluster C, which contained all AM and control chickens of 14, 21, and 35 days old. This difference in distribution of AM and control chickens over cluster A and B in the 1st week of life indicates an accelerated maturation of caecal microbiota profiles for AM chickens. In contrast, clustering for the ileal content profiles only showed an effect of age, with cluster D dominated by 3- and 7-day-old chickens of both treatments, and cluster E by chickens of 14, 21, and 35 days old of both treatments (Figure 3B). The relative microbial abundance of the clusters observed in the caecal content was analyzed and although PBS and AM chickens varied in their relative abundance of microbial families, PBS and AM chickens can be part of the same cluster based on relative abundance of genera (Figure 3C).

### AM Treatment Affects Presence of NK Cell Subsets and Their Activation

**Figure 4:**
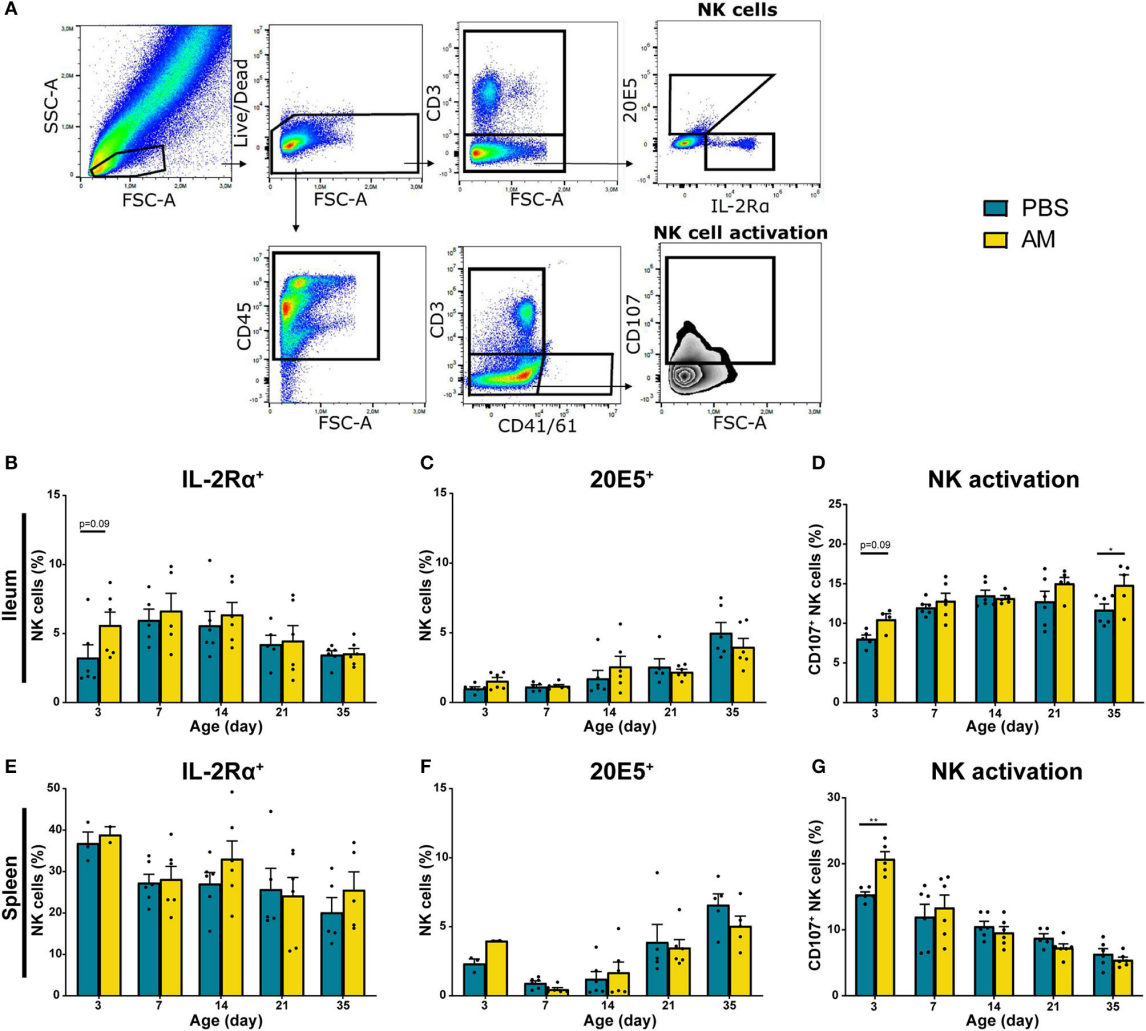
Effect of adult microbiota (AM) on NK cells in broiler chickens. **(A)** Gating strategy after isolation of lymphocytes from IEL to analyze NK cell subsets and activation. **(B,E)** Percentages of NK cell subsets by characterization of surface markers IL-2Rα, **(C,F)** 20E5 during aging in **(B–D)** IEL and **(E–G)** spleen. **(D,G)** Percentages of NK cell activation during aging as assessed by measuring the surface marker CD107. Mean + SEM of chickens is shown (*n* = 6), however, chickens were excluded from analysis when numbers of events acquired in the gate of interest were < 100. Statistical significance is indicated as *p < 0.05 and **p < 0.01.

Possible differences in subsets and activation of intestinal NK cells from AM and control chickens were determined. Local effects of AM inoculation on intestinal NK cells were compared to systemic effects measured in spleen and blood. Within the live lymphocytes, the CD3 negative IL-2Rα^+^ or 20E5^+^ NK cells were quantified (Figure 4A). In parallel, NK cell activation was determined by analysis of enhanced CD107 surface expression on CD3 negative and CD41/61 negative cells. At day 3, the percentage of intestinal IL-2Rα^+^ NK cells tended to be higher in AM chickens (5.61 ± 0.95%) compared to controls (3.25 ± 0.93%, *p* = 0.09, Figure 4B). No differences between treatment groups were observed in intestinal 20E5^+^ NK cells (Figure 4C). Increased CD107 expression on intestinal NK cells was observed at day 3 in AM chickens (10.52 ± 0.70%), when compared to controls (8.07 ± 0.47%, *p* = 0.06, Figure 4D). At day 35, an increase in activation of intestinal NK cells was observed in AM chickens (14.86 ± 1.27%) compared to the controls (11.71 ± 0.75%, *p* = 0.04, Figure 4D). No differences between treatment groups were observed in CD107 expression of intestinal NK cells at other ages (Figure 4D).

Relative numbers of IL-2Rα^+^ and 20E5^+^ NK cells in spleen and blood were similar in both treatment groups (Figures 4E,F, Supplementary Figures 3A,B). However, NK cell activation was significantly increased in splenic NK cells in 3-day-old AM chickens (20.74 ± 1.10%) compared to controls (15.35 ± 0.40%, *p* = 0.004, Figure 4G). No difference in CD107 surface expression on blood-derived NK cells was found between treatment groups (Supplementary Figure 3C). Furthermore, AM inoculation did not affect total lymphocyte numbers in the intestine, spleen and blood (Supplementary Figures 4A,E,I). In addition to the percentages of the different NK subsets, absolute numbers were determined. Similar trends were observed in absolute number of IL-2Rα^+^, 20E5^+^, and CD107^+^ NK cells although the differences between treatments were less pronounced (Supplementary Figure 4).

### AM Treatment Affects Intestinal Cytotoxic CD8αα T Cells in 14- and 21-Day-Old Chickens

**Figure 5:**
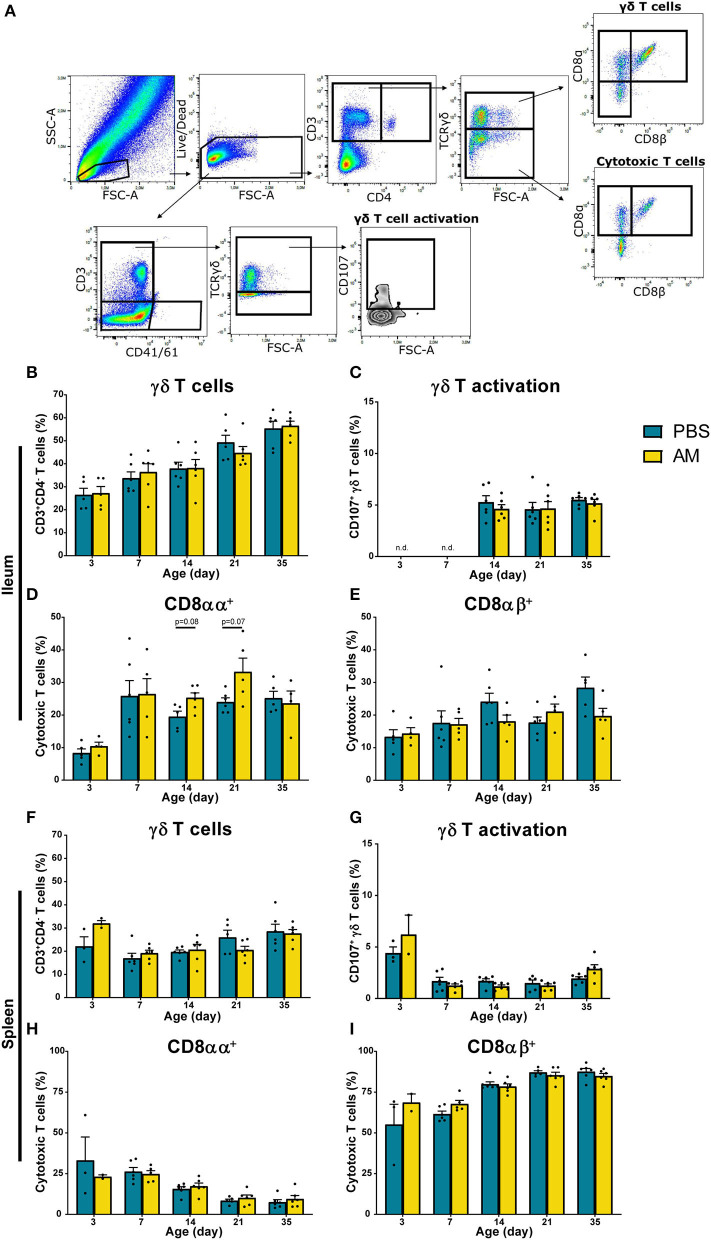
Effect of adult microbiota (AM) on T cells in broiler chickens. **(A)** Gating strategy after isolation of lymphocytes from IEL to analyze T cell subsets and γδ T cell activation. **(B,F)** Percentages of total γδ T cells, subsets (data not shown) and **(C,G)** γδ T cell activation by characterization of surface markers TCRγδ and CD107 during aging in **(B–E)** IEL and **(F–I)** spleen. **(D,H)** Percentages of cytotoxic T cell subsets using the surface markers CD8αα and **(E,I)** CD8αβ during aging. Mean + SEM of chickens is shown (*n* = 6), however, chickens were excluded from analysis when numbers of events acquired in the gate of interest were <100.

In addition to NK cell subsets and NK cell activation, effects of AM inoculation on presence and function of γδ T cells and presence of cytotoxic CD8^+^ T cells were studied. Within the CD3^+^ and CD4^−^ lymphocytes, both TCRγδ^+^ and TCRγδ^−^ cell populations were analyzed for CD8αα and CD8αβ expression (Figure 5A). In parallel, activation of γδ T cells was determined at day 7, 14, and 21 by analyzing increased surface expression of CD107 on CD3^+^CD41/61^−^ TCRγδ^+^ cells (Figure 5A). No differences between AM and control chickens were observed in the percentage of intestinal γδ T cells (Figure 5B), CD8^−^, CD8αα^+^, and CD8αβ^+^ gamma delta subsets (data not shown) and activation of γδ T cells (Figure 5C). The percentage of intestinal CD8αα^+^ T cells tended to be higher in 14- (25.3 ± 1.5%) and 21-day-old AM chickens (33.2 ± 4.2%) compared to controls (19.5 ± 1.7%, *p* = 0.08 and 24.0 ± 1.3%, *p* = 0.07, respectively, Figure 5D). No differences between groups were found at any age in the percentages of intestinal CD8αβ^+^ T cells (Figure 5E). Furthermore, no differences between AM and control chickens were observed in the percentage of γδ T cells (Figure 5F, Supplementary Figure 3D), subsets (data not shown), γδ T cell activation (Figure 5G, Supplementary Figure 3E) and cytotoxic T cells in spleen and blood (Figures 5H,I, Supplementary Figures 3F,G). Absolute numbers of these parameters were investigated and did not show any differences between AM and control chickens, although an increase in numbers of both treatments was observed with age (Supplementary Figure 5).

### Association Between Caecal Microbiota Clusters and Immune Cells

Clustering of the caecal content profiles suggests that AM chickens showed an earlier maturation of caecal microbiota profiles compared to controls. Also, differences in IL-2Rα^+^ NK cells, NK cell activation and CD8αα T cells were observed between AM chickens compared to the controls. To assess a possible relationship between the observed differences in the microbial development between treatments and the detected differences in immune parameters, we used the previously identified DMM clusters to test for correlations between the caecal microbiota profiles (i.e., stages of successive microbiota maturation) and immune parameters.

**Figure 6:**
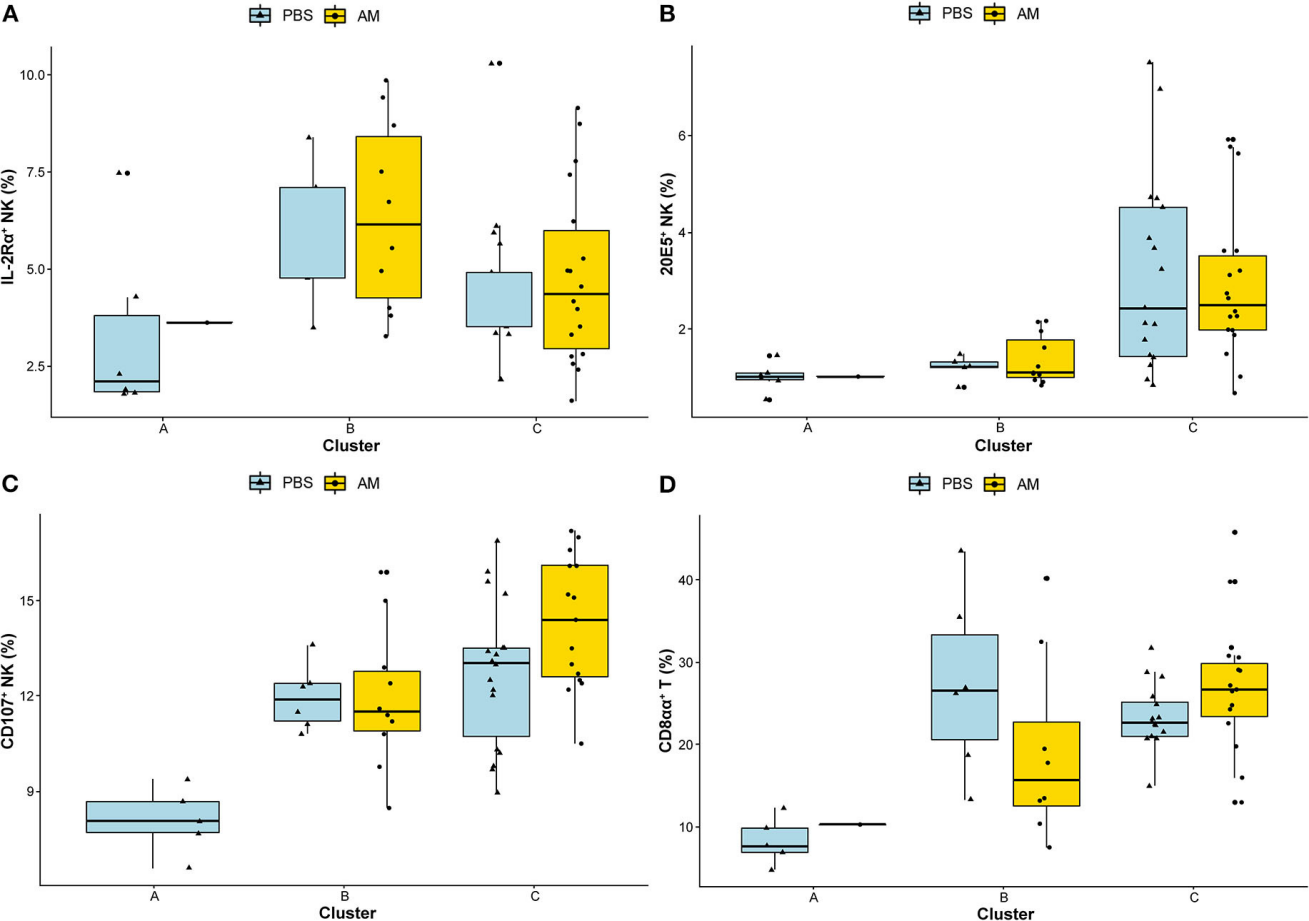
Associations between caecal microbiota clusters and immune cells. Associations between the identified DMM clusters of caecal microbiota composition and relative numbers of intestinal NK cell subsets **(A)** IL-2Rα^+^, **(B)** 20E5^+^, **(C)** CD107^+^, and **(D)** cytotoxic CD8αα^+^ T cells were analyzed using Wilcoxon rank-sum test. Adjusted *p*-values (<0.05) were corrected for multiple testing with BH.

Clusters A, B, and C were based on relative abundance of genera present in the caecal microbiota of chickens and represent different stages during the early life development of caecal microbiota. Correlations to relative and absolute numbers of IL-2Rα^+^, 20E5^+^, CD107^+^ NK cells, and cytotoxic CD8aa^+^ T cells in the ileum were investigated. The percentage of intestinal IL-2Rα^+^ NK cells was higher in cluster B compared to cluster A (*p* = 0.026, Table 2), and compared to cluster C (*p* = 0.044, Table 2) regardless of treatment (Figure 6A). The percentage of IL-2Rα^+^ NK cells in cluster C tended to be higher compared to cluster A, but this was not significant (*p* = 0.068, Table 2, Figure 6A). Relative numbers of intestinal 20E5^+^ NK cells were similar between clusters A and B and highest in cluster C (Figure 6B, Table 2). Relative numbers of intestinal CD107^+^ NK cells were highest in cluster C and lowest in cluster A (Table 2, Figure 6C). Within cluster C, the percentage of CD107^+^ NK cells tended to be higher in AM chickens (Figure 6C). Relative numbers for intestinal cytotoxic CD8αα^+^ T cells were higher in cluster B and C compared to cluster A and did not differ between cluster B and C (Figure 6D, Table 2). Similar correlations were observed between clusters and absolute numbers of intestinal 20E5^+^, CD107^+^ NK cells and cytotoxic CD8αα^+^ T cells (Table 2).

**Table 2:** Statistical differences in relative (%) and absolute (cells/mg) numbers of intestinal immune cells between caecal microbiota clusters.

**Immune cells**	**Cluster A vs. B**	**Cluster B vs. C**	**Cluster A vs. C**
IL-2Rα^+^ NK (%)	**0.026**	**0.044**	0.068
20E5^+^ NK (%)	0.124	**3.0e**^**−4**^	**0.001**
CD107^+^ NK (%)	**0.003**	**0.020**	**0.001**
CD8αα^+^ T (%)	**0.001**	0.254	**4.1e**^**−4**^
IL-2Rα^+^ NK (cells/mg)	**0.011**	0.051	**2.7e**^**−4**^
20E5^+^ NK (cells/mg)	**0.039**	**5.3e**^**−7**^	**2.1e**^**−6**^
CD107^+^ NK (cells/mg)	0.398	**4.0e**^**−6**^	**1.1e**^**−4**^
CD8αα^+^ T (cells/mg)	**0.008**	**9.5e**^**−6**^	**6.4e**^**−4**^

*Significant differences are indicated in bold*.

These results indicate significant associations between caecal microbiota clusters and subsets of intestinal immune cells.

## Discussion

In this study, we aimed to induce an alteration in the intestinal microbiota shortly after hatch by administration of adult-derived microbiota, and compared presence and function of NK cells, as representatives of developing innate immunity, to those of non-inoculated controls. We hypothesized that early exposure to adult-derived microbiota would accelerate intestinal microbiota colonization and affect subsets and activation of intestinal NK cells. Our results indicate that the inoculation with the adult-derived microbes mostly affected the early development of the caecal microbiota, and induced an earlier maturation of caecal microbiota compared to control broiler chickens. This development was paralleled by an increase in intestinal IL-2Rα^+^ NK cells and enhanced activation of NK cells early in life and CD8αα^+^ T cells later in life.

The AM inoculation delivered immediately after hatch successfully altered intestinal microbiota composition, especially in the 1st week of life, but did not permanently influence the diversity of caecal microbiota. In addition, with respect to the genera found in the AM product, a higher relative abundance was only found shortly after inoculation. More specifically, a higher relative abundance in AM chickens was found for 10 of the 24 genera in the inoculum on day 1, but this quickly declined to two genera by the end of the 1st week. These findings are in line with previous studies with the same product: inoculation with Aviguard *in ovo* enhanced development of intestinal microbiota of broiler chickens and increased diversity and reduced the abundance of *Enterobacteriaceae* (22). Similar to our study, not all genera present in the inoculum permanently colonized the intestine; they were assumed to have been transient colonizers facilitating the development of a complex microbiota by temporarily altering the microenvironment (22). Similar observations have been reported for 1-day-old laying hens inoculated with Aviguard. Not all bacteria of the product, nor of the mother hen, were effectively transferred to the chickens' gut, but compared to controls, caecal microbiota enriched for the phyla *Bacteroidetes* and *Actinobacteria* was observed within a week in both Aviguard treated chickens and in chickens naturally exposed to a mother hen (55).

Like chickens hatched in commercial hatcheries, the control chickens in our study were gradually exposed to microbiota in the hours and days after hatch from different sources, such as the housing environment, litter, feed, and water. This colonization was delayed compared to the chickens inoculated with AM directly after hatch, as indicated by the clustering of caecal content profiles of 3-day-old controls with 1-day-old AM inoculated chickens, and of 7-day-old controls with 3-day-old AM chickens. This accelerated maturation of caecal microbiota composition has not only been observed in Aviguard studies (22, 55), but also in a study in which topical spray treatment of eggs with adult caecal content significantly altered broiler chicken microbiota immediately after hatch, and accelerated the normal microbiota development (69). As in our study, the effect on the caecal microbiota was highest at 3 days of age, and diminished over time (69). In contrast, swabbing of the egg surface once during incubation with diluted adult caecal content did not lead to significant differences in alpha diversity nor in the pattern of bacterial colonization between treated and control broiler chickens (70). This difference may be a result of the egg inoculation technique, suggesting that perhaps a lower number of spores and vegetative cells was applied to the eggshell in the latter study.

Although many of the available poultry microbiota studies have focused on broiler chickens, its relation with the innate immune system has not previously been elaborately investigated. We observed an increase in IL-2Rα^+^ NK cells and activation of NK cells within the 1st days of life, together with an increase in relative numbers of cytotoxic CD8αα^+^ T cells from day 14 onwards in chickens that were inoculated with AM.

The increased NK cell activation observed in AM chickens may suggest a mildly increased cytotoxic capacity against potential pathogens, as the CD107 expression can increase up to 30% upon viral infections (33), which is more than 2-fold higher than the NK cell activation observed in this study. This result is in line with the observed increase in IL-2Rα^+^ NK cells in this study. Studies in humans have shown that increased IL-2Rα expression is associated with an early stage of NK cell activation (31), and this was also observed in chickens (32, 71, 72). In addition to the local effect on NK cell activation, our observation of increased splenic NK cell activation in 3-day-old AM chickens also indicates there is a systemic effect. No effects of AM inoculation on immune cells in the blood were observed.

The observed differences between AM and control chickens with respect to immune parameters suggest an interaction between microbial and immune development. This was further substantiated by the significant associations between IL-2Rα^+^ NK cells, CD107^+^ NK, and CD8αα^+^ T cells and caecal microbiota clusters: cluster A includes chickens with a starting microbiota, cluster B chickens in the middle of the maturation process and cluster C chickens with a more matured successive microbiota composition from day 14 onwards. These clusters follow the successional patterns of microbiota development as previously described for broiler chickens, with bacterial community richness increasing rapidly over time and stabilizing from day 14 onwards (5–7). Our analyses showed that cluster B was associated with an increase in IL-2Rα^+^ NK cells and an enhanced NK cell activation regardless of treatment. This suggests that the accelerated microbiota colonization due to AM inoculation affected the development of NK cells locally and systemically. Interestingly, the IL-2Rα^+^ NK cell subset was higher in relative numbers in cluster B compared to the starting microbiota cluster A, but subsequently decreased in relative numbers in the more mature microbiota cluster C. The 20E5^+^ NK cell subset and NK cells that express CD107 further increased in relative numbers between cluster B and cluster C. This fits with the observation in mammals that an increase in IL-2Rα expression is associated with an early stage of NK cell activation, which is followed by enhanced NK cell mediated killing. Cluster C was associated with an increased relative number of intestinal cytotoxic CD8αα^+^ T cells. As the caecal microbiota in this cluster shows a matured composition similar in AM and control chickens of the same age, this suggests that early life inoculation with AM also affected the adaptive immune development in the intestine.

Although these results indicate associations between early life microbiota colonization and immune system development, the data from this study cannot elucidate exactly how these processes are related. As has been shown in humans and mice, microbiota can signal to immune cells in various ways either locally or systemically (46, 73). Locally, microorganisms interact directly with NK cells via TLRs and NCRs resulting in cytokine production by NK cells, and indirectly via cytokine production of resident myeloid or epithelial cells that consequently affect NK cell responses (46, 74). Systemically, microbiota can induce instructive signals to non-mucosal antigen-presenting cells and by producing among others IL-15, TNFα and IFN, subsequently prime optimal splenic NK cell responses (73). Since chicken NK cells have been shown to express TLRs (75) and NCRs (76, 77), the interactions between microbiota and NK cells probably follow similar routes to those in humans and mice.

In mammals, specific commensal bacterial strains have been linked to modulation of NK cells. Several reports established that bacteria within the *Lactobacillus* genus can induce IFNγ and cytotoxicity responses in intestinal NK cells as a result of IL-12 production by dendritic cells after TLR engagement with bacteria (43, 78, 79). Furthermore, *Bacteroides fragilis* can stimulate innate and adaptive immune pathways directly through TLR signaling and indirectly by inducing cytokine production (80). Although we did observe significant differences in the relative abundance of genera between AM and control chickens at day 1 and 3, we cannot pinpoint a specific genus responsible for the observed effect on NK cells. Interestingly, the genus *Bacteroides* showed a significantly higher prevalence and relative abundance in 3- and 7-day-old AM chickens and the genus was absent in control chickens of similar age. This could suggest that the observed effects on NK cells in 3-day-old AM chickens may be linked to a higher presence of *Bacteroides* bacteria as shown previously (80). We did not find differences in the prevalence of *Lactobacillus* bacteria due to AM inoculation. Other genera that showed significant differences in their prevalence and/or relative abundance between AM and control chickens at 1 and 3 days of age have not been described as specifically interacting with NK cells.

In addition, microbiota has been shown in mice and humans to interact directly with γδ T cells, and increased frequencies of CD8^+^ γδ T cells and γδ T cell activation were observed during intestinal inflammation (51, 81). Under non-inflammatory conditions similar to those of our study, application of adult caecal content on eggs altered and accelerated the microbiota of 3-day-old chickens but did not affect γδ T cells in caecal tonsils (69). Furthermore, AM inoculated chickens in our study showed an increased presence of intestinal CD8αα^+^ T cells at 2 and 3 weeks of age. Although in previous studies with mice no CD8αα and CD8αβ subsets were investigated, microbiota was shown to have direct (53) and indirect (82) effects on cytotoxic T cells, as IFNγ production was induced.

Further research including challenge models is needed to answer the question if chickens with an accelerated maturation of intestinal microbiota and enhanced NK cell responses early in life are indeed more resilient against infections.

Interestingly we observed a relation between changes in caecal microbiota and intestinal NK cell responses. It would have been highly interesting to investigate the interaction between immune system and microbiota at caecum level, but unfortunatelythis was not possible since only few NK cells can be obtained from the caecum in young chickens (83, 84). Although we set out to analyze the relation between NK cells and microbiota composition in the ileum, we did not observe differences between treatment groups at any age in the phylogenetic diversity of ileal microbiota nor in the relative abundances of genera. Not being able to show a difference at ileal level, especially considering the relatively small number of chickens at each time point, was not surprising, and exactly the reason why we also collected caecal content. Nevertheless, the shift in microbiota composition as measured in the caeca showed that the AM treatment has successfully affected microbiota development in parts of the intestinal tract. For the AM treatment to be able to alter caecal microbiota composition, the microbiota of the AM product at least must have passed, and to some extent may have colonized upstream parts of the intestinal tract as well, albeit not inducing a measurable shift in microbiota composition in ileum. Therefore, we expect that the observed effects on ileal NK cells are associated with the AM treatment.

In conclusion, our study showed a relation between an accelerated maturation of intestinal microbiota and the enhanced NK cell response early in life. This interaction between microbiota and the developing innate immune system indicates possibilities in developing strategies to improve health and resilience of broiler chickens. One such possibility is through feed interventions or the use of products with adult-derived microbiota directly after hatch, both of which can affect microbiota composition and may accelerate microbiota maturation. Consequently, this can strengthen the innate immune system, conferring direct protective effects early in life as well as influencing adaptive immunity later in life. The combination of a well-developed microbiota and immune system may result in more robust broiler chickens with higher resilience against health challenges, such as disturbances in gut health and invading pathogens. Future research including challenge studies are warranted to test this hypothesis.

## Data Availability Statement

Raw sequence data were deposited into the Sequence Read Archive (SRA) at NCBI under accession number PRJNA670739.

## Ethics Statement

The animal study was reviewed and approved by the Dutch Central Authority for Scientific Procedures on Animals and the Animal Experiments Committee.

## Author Contributions

NM, JK, FV, DL, JO, HS, JS, VR, and CJ contributed to the conception, design of the study, drafting, and critically revising it for important intellectual content. NM, JK, FV, DH, and CJ contributed to acquisition of data. NM and JK performed the analysis of data. FV and CJ supervised the work. All authors approved the final version to be submitted.

## Conflict of Interest

The authors declare that the research was conducted in the absence of any commercial or financial relationships that could be construed as a potential conflict of interest.
