# Supplementary material for: Early Life Inoculation With Adult-Derived Microbiota Accelerates Maturation of Intestinal Microbiota and Enhances NK Cell Activation in Broiler Chickens
Source: Front Vet Sci. 2020 Nov 19;7:584561. doi: 10.3389/fvets.2020.584561 (PMC7710667; doi:10.3389/fvets.2020.584561)
Supplement: Supplementary file 1 [file Data_Sheet_1.docx]

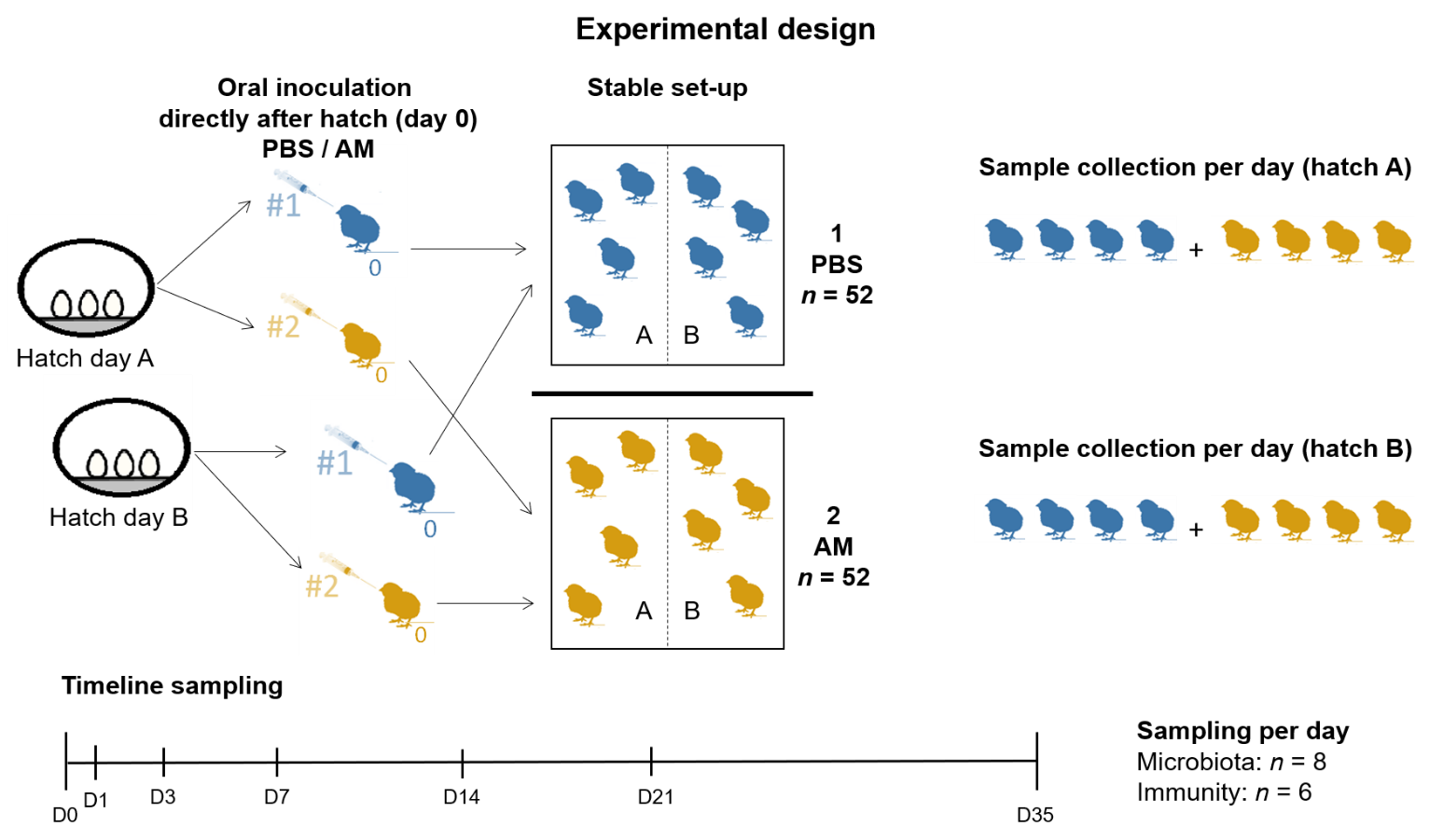


**Supplementary Figure 1**. Experimental design of the study. The Ross broiler chickens hatched at different days. Immediately after hatch (day 0), chicks were orally inoculated with either PBS (*n* = 52) or AM (*n* = 52) and transferred to the corresponding floor pen in the stable. Floor pens of PBS- and AM-inoculated chickens were separated by a wall in between. Four chickens per treatment group were collected per sample day. The timeline for sampling is shown in the lower part of the figure; per sampling day, eight chickens were used for microbiota analysis and six chickens were used for immunity analysis.


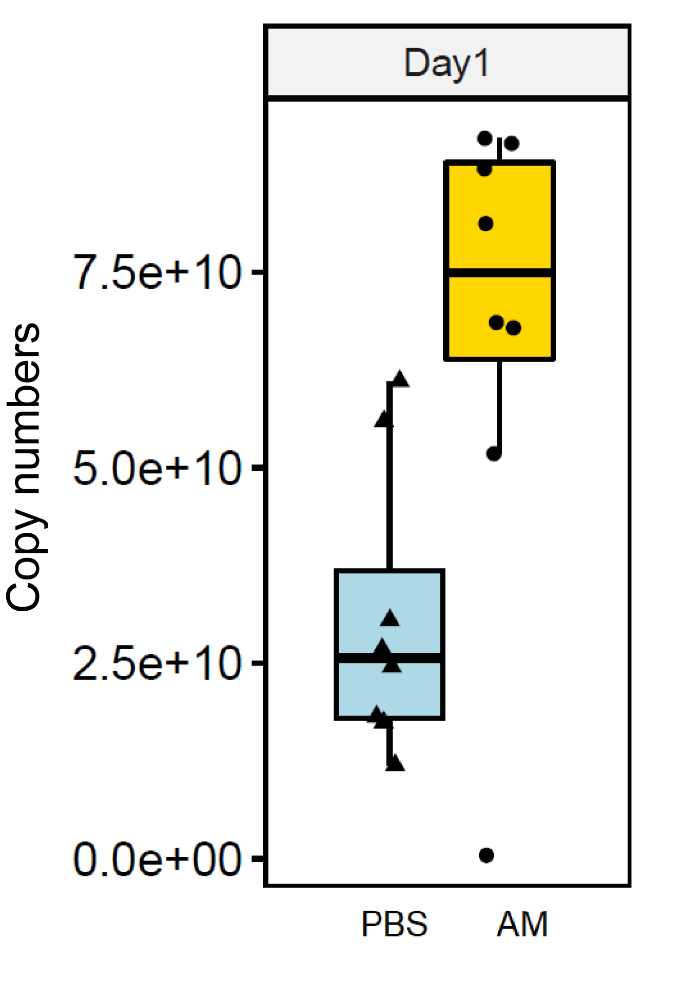


**Supplementary Figure 2**. Visualization of 16S rRNA gene count data in the caecum of 1-day-old chickens as determined by qPCR. Significant difference (*p* < 0.05, Kruskal-Wallis test) is indicated in gene copy numbers between control chickens (PBS, blue) and AM chickens (yellow). *n* = 8 chickens per treatment, whiskers show 95% interval, box 50% interval.


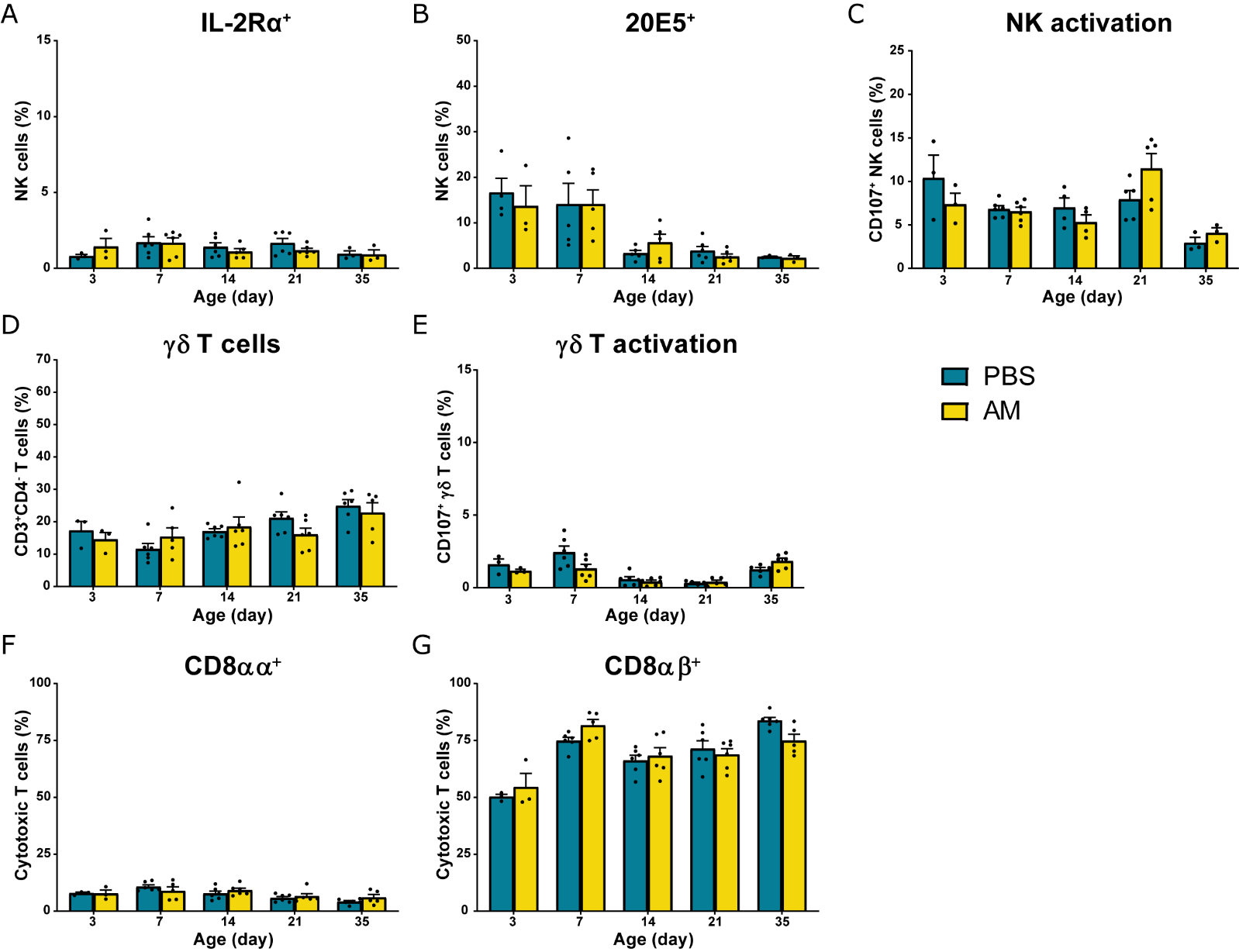


**Supplementary Figure 3.** Effect of adult microbiota (AM) on NK and T cells in blood of broiler chickens. (**A**) Percentages of NK cell subsets characterized by surface markers IL-2Rα and (**B**) 20E5 during aging in blood. (**C**) Percentages of NK cell activity during aging as assessed by measuring the surface marker CD107. (**D**) Percentages of total γδ T cells and (**E**) γδ T cell activation by characterization of surface markers TCRγδ and CD107, respectively. (**F**) Percentages of cytotoxic T cell subsets using the surface markers CD8αα and (**G**) CD8αβ during aging in blood. Mean + SEM of chickens is shown (*n* = 6), however, chickens were excluded from analysis when numbers of events acquired in the gate of interest were < 100.


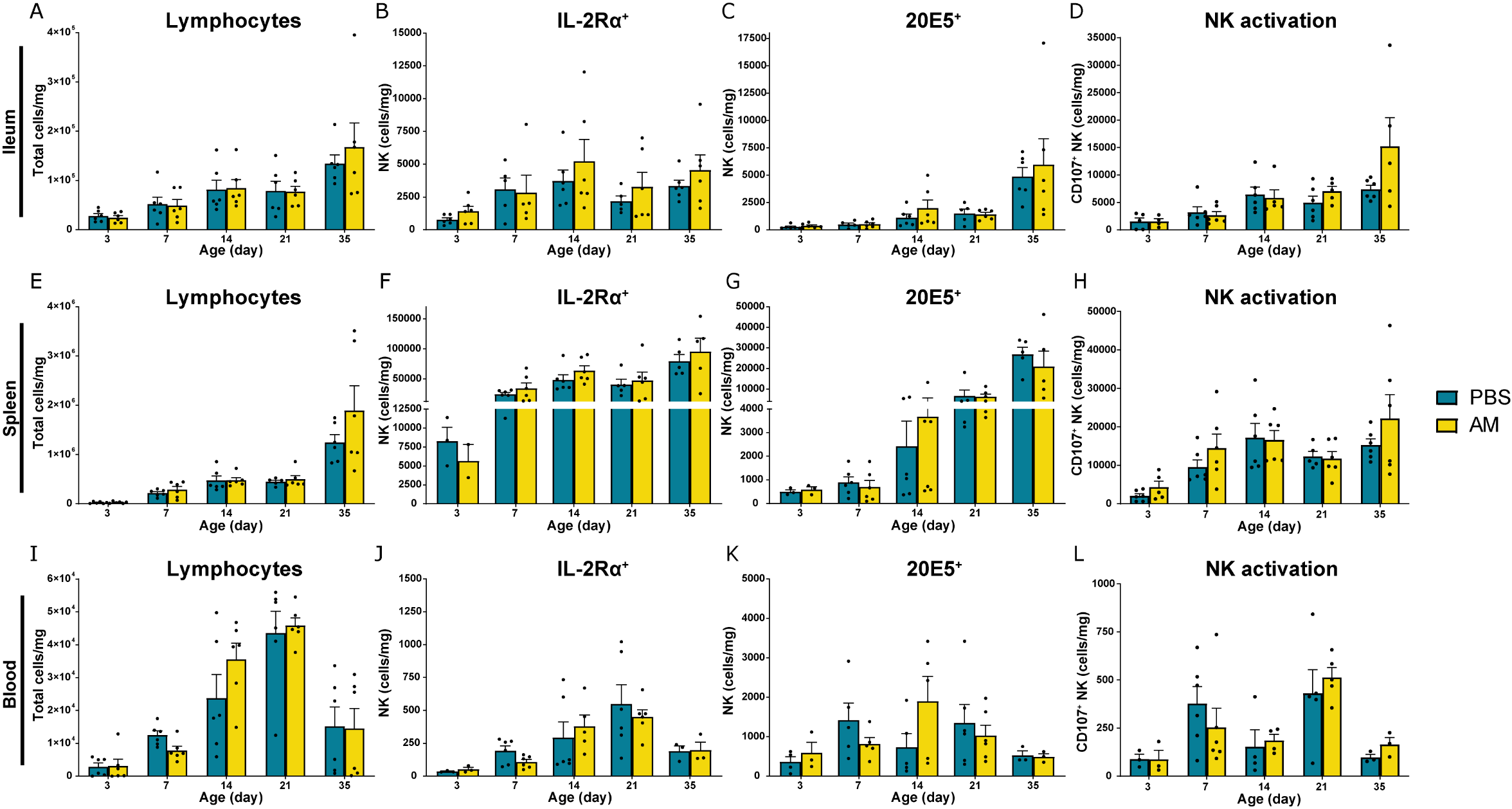


**Supplementary Figure 4.** Effect of adult microbiota (AM) on NK cell numbers in broiler chickens. **(A)** Total cell numbers per mg organ isolated from IEL, **(E)** spleen and **(I)** blood. **(B,F,J)** Absolute NK cell numbers for subsets by characterization of surface markers IL-2Rα and **(C,G,K)** 20E5 during aging in **(A-D)** IEL, **(E-H)** spleen and **(I-L)** blood. **(D,H,L)** Cell numbers for NK cell activation during aging as assessed by measuring the surface marker CD107. Mean + SEM of chickens is shown (*n* = 6), however, chickens were excluded from analysis when numbers of events acquired in the gate of interest were < 100.


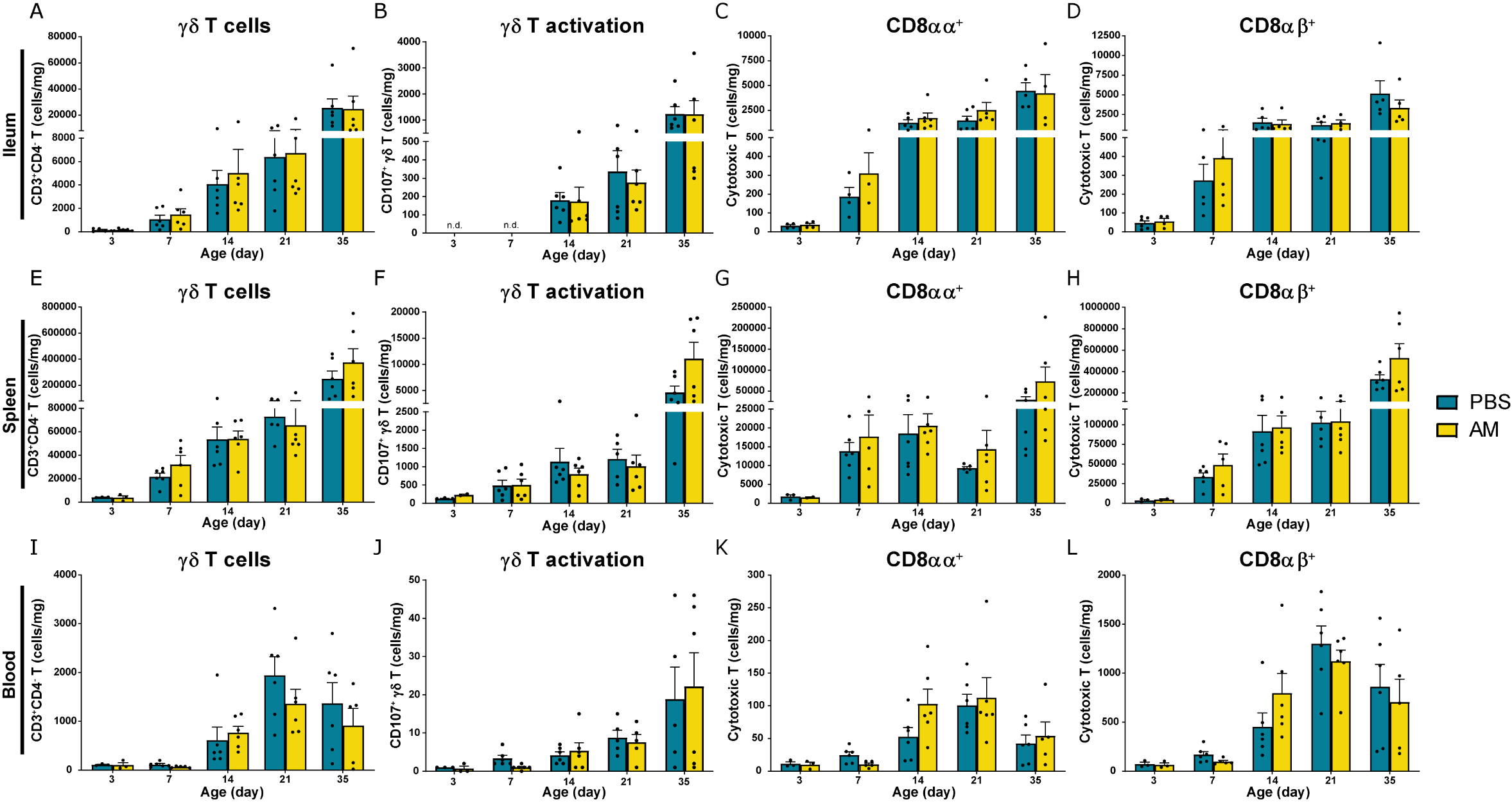


**Supplementary Figure 5.** Effect of adult microbiota (AM) on T cell numbers in broiler chickens. (**A**,**E**,**I**) Absolute cell numbers of γδ T cells and (**B**,**F**,**J**) γδ T cell activation by characterization of surface markers TCRγδ and CD107, respectively, during aging in (**A**-**D**) IEL, (**E**-**H**) spleen and (**I**-**L**) blood. (**C**,**G**,**K**) Absolute cell numbers of cytotoxic T cell subsets by characterization of surface markers CD8αα and (**D**,**H**,**L**) CD8αβ during aging. Mean + SEM of chickens is shown (*n* = 6), however, chickens were excluded from analysis when numbers of events acquired in the gate of interest were < 100.
